# Synthesis, Properties, and Selected Technical Applications of Magnesium Oxide Nanoparticles: A Review

**DOI:** 10.3390/ijms222312752

**Published:** 2021-11-25

**Authors:** Jaroslav Hornak

**Affiliations:** Department of Materials and Technology, Faculty of Electrical Engineering, University of West Bohemia, 301 00 Pilsen, Czech Republic; jhornak@fel.zcu.cz; Tel.: +420-37763-4530

**Keywords:** magnesium oxide, synthesis, bottom-up, crystallite size, nanomaterials, structural properties, dielectric properties, electrotechnical applications

## Abstract

In the last few decades, there has been a trend involving the use of nanoscale fillers in a variety of applications. Significant improvements have been achieved in the areas of their preparation and further applications (e.g., in industry, agriculture, and medicine). One of these promising materials is magnesium oxide (MgO), the unique properties of which make it a suitable candidate for use in a wide range of applications. Generally, MgO is a white, hygroscopic solid mineral, and its lattice consists of Mg${}^{2+}$ ions and O${}^{2-}$ ions. Nanostructured MgO can be prepared through different chemical (bottom-up approach) or physical (top-down approach) routes. The required resultant properties (e.g., bandgap, crystallite size, and shape) can be achieved depending on the reaction conditions, basic starting materials, or their concentrations. In addition to its unique material properties, MgO is also potentially of interest due to its nontoxicity and environmental friendliness, which allow it to be widely used in medicine and biotechnological applications.

## 1. Introduction

Nanostructured materials, and nanotechnology in general, have received considerable attention in the last few decades, with significant overlap in the techno-economic sector [1,2,3]. The increased interest stems mainly from the fact that reducing materials to the nanoscale can lead to them having unique properties that are not possible in the bulk material at the micro- and macroscales [4]. In general, there is a fundamental consensus that nanomaterials are materials whose dimensions do not exceed 100 nm [5]. Nanomaterials can be further classified [6,7] based on (i) dimensionality (0D–3D), (ii) morphology (e.g., nanospheres, nanotubes, and nanowires), (iii) state (e.g., isometric, suspension, and agglomerates), or (iv) chemical composition (e.g., organic, inorganic, single component, and composites).

**Figure 1:**
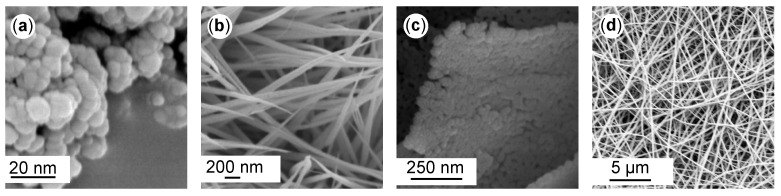
Different material structures. (**a**) 0D: SiO${}_{2}$ nanoparticles [16]; (**b**) 1D: CoO nanowires [17]; (**c**) 2D: ZnO–NiO nanosheets [18]; and (**d**) 3D: PA6 nonwoven structure [19]. All reproduced from articles distributed under the terms and conditions of the Creative Commons Attribution (CC BY) license.

Selected examples with dimensions varying from 0D to 3D and with different morphologies are presented in Figure 1. In the case of nanomaterial classification based on chemical composition, a primary division into organic and inorganic can be considered [8]. In the first case, organic-based nanostructures are more environmentally friendly materials (e.g., ferritin, liposomes, micelles, and dendrimers) that, due to their nature, are more suitable, e.g., for drug delivery [9].

The category of inorganic materials can be further differentiated into metal-based and metal oxide-based materials [10]. Metal-based nanomaterials are synthesized using metallic materials, such as Au, Cu, Se, and Ag [11], and they find applications in areas, such as radiotherapy enhancement, gene delivery, or thermal ablation [12]. Metal oxide-based materials are, as the name suggests, formed as a product of the oxidation reaction of a metallic material in the presence of oxygen [13].

The most important representatives of the single metal oxide group are silicon dioxide (SiO${}_{2}$), ferric oxide (Fe${}_{2}$O${}_{3}$), zinc oxide (ZnO), titanium dioxide (TiO${}_{2}$), and magnesium oxide (MgO) [14]. Due to their unique properties, these materials find applications in areas, such as medicine, agriculture, information technology, electronics, energy, and environmental protection [15].

**Figure 2:**
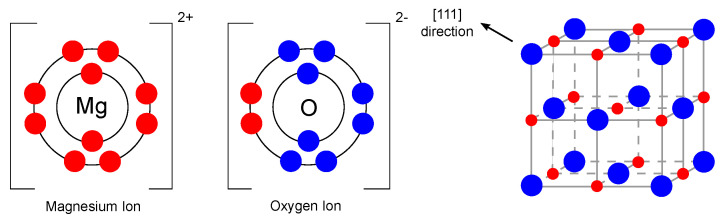
Electronic configuration of Mg${}^{2+}$ and O${}^{2-}$ ions and crystal structure of MgO (redrawn and adapted from [23,25]).

As mentioned earlier, one of the promising materials belonging to the single metal oxide group is magnesium oxide. Magnesium oxide, also referred to as periclase [20], is an inorganic material with a molar mass of 40.31 g/mol [21] and a density of 3.58 g/cm${}^{3}$ [22]. Its empirical formula is MgO, and its lattice consists of Mg${}^{2+}$ ions and O${}^{2-}$ ions linked by an ionic bond in a 1s${}^{2}$2s${}^{2}$p${}^{6}$ and 1s${}^{2}$2s${}^{2}$p${}^{6}$ configuration, which means that the d-orbitals are empty in this case [23]. The magnesium oxide structure is of the rock-salt type (lattice parameter 4.21 Å [24]). In general, it consists of two intersecting Mg and O lattices that are offset relative to each other by 0.5 of the body diagonal. The electronic configuration and crystal structure are shown in Figure 2.

**Figure 3:**
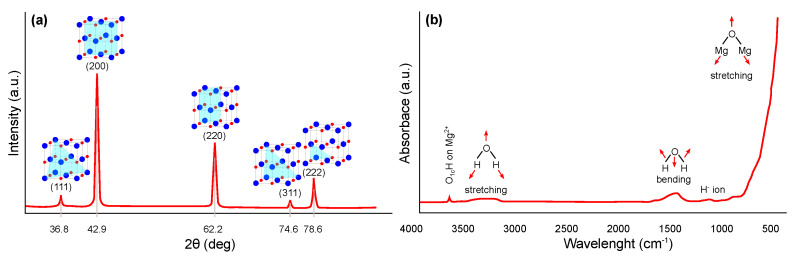
Structural characterization of magnesium oxide: (**a**) XRD pattern and (**b**) FTIR spectra (redrawn and adapted from the results presented in [26,27,28,29,30,37,38,39,40]).

The crystal structure of magnesium oxide can be suitably characterized using X-ray diffraction (XRD) (JCPDS Standard No. 87-0653), where significant peaks can be assigned close to the 2θ angle values of 36.8, 42.9, 62.2, 74.6, and 78.6°, which can be indexed to the lattice planes (111), (200), (220), (311), and (222), respectively. The typical pattern is shown in Figure 3a, whereby similar results have been widely published [26,27,28,29,30]. The Scherrer [31] equation is most commonly used in the literature to determine the crystallite size of synthesized nanostructures from XRD results.

However, the limiting factor in terms of using the Scherrer equation is the average crystallinity size up to *ca.* 200 nm [32]. This is due to the fact that broadening of the diffraction peak decreases with increasing crystallite size [33]. Thus, it is difficult to separate the broadening of the peak due to crystallite size from the broadening due to other factors (e.g., shape and size distributions of the crystallites).

This equation also does not provide information about the lattice microstructure, i.e., the internal deformation that is obtained in nanocrystals due to point defects, grain boundaries, triple transitions, and stacking faults [34]. Therefore, to acquire a more complete microstructural description, the Williamson–Hall (W-H) [35] equation can be used. This approach is based on the simplified integral breadth method, where both size-induced and strain-induced broadening are deconvoluted by considering the peak width as a function of 2θ [36].

Fourier transform infrared spectroscopy (FTIR) can also be used for the identification and structural characterization of magnesium oxide [37,38,39,40]. The most common interpretation is in the form of transmittance or absorbance, both depending on the wavelength of the incident radiation. A typical FTIR spectrum in the absorbance mode for magnesium oxide is shown in Figure 3b. A narrow and weak peak around 3700 cm${}^{-1}$ indicates the presence of mono-coordinated hydroxyl (–OH) groups. A broader peak centered around 3724 cm${}^{-1}$ may also occur, indicating the presence of three coordinated –OH groups. Next broader peak between 3200 and 3650 cm${}^{-1}$ indicates H-bonded –OH groups or their stretching vibrations, respectively. Close to 1430 cm${}^{-1}$, the bending vibration of the –OH groups of physically adsorbed water molecules may be visible. Finally, a strong peak centered around 470 cm${}^{-1}$ represents the stretching vibration of Mg–O.

In addition to the above, ultraviolet–visible spectroscopy (UV–Vis) can be used to characterize MgO, particularly to determine the bandgap energy [41,42,43]. For this purpose, absorbance spectra and Tauc plots are used. The photoluminescence method (PL) [44,45] is also frequently used to determine the bandgap width.

**Figure 4:**
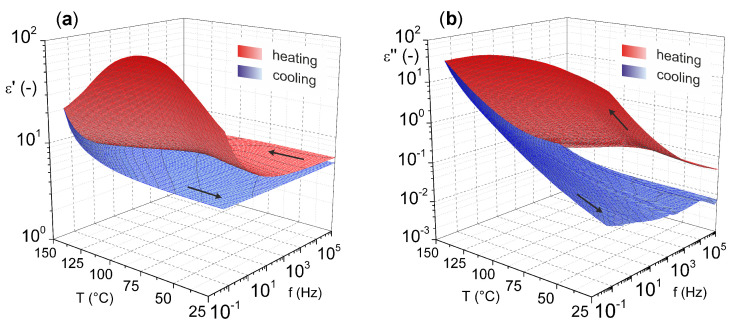
Temperature–frequency dependence of complex permittivity: (**a**) real part and (**b**) imaginary part (reprinted from article [46] distributed under the terms and conditions of the Creative Commons Attribution (CC BY) license).

For a more comprehensive view on magnesium oxide, the results of our previous study [46] characterizing the dielectric parameters of magnesium oxide (MgO nanoparticles pressed into pellet) as a function of temperature and frequency are shown in Figure 4. These results illustrate the relatively good temperature stability of the relative permittivity. Nevertheless, increases are more pronounced in the lower frequency and higher temperature regions. This statement is especially valid for the cooling mode (from higher to lower temperatures), because in the heating mode (from lower to higher temperatures), the effect of weakly bound water molecules on the surface of the material is evident.

The presence of water molecules influences both investigated parameters. In this case, an important role is played by dehydration of the material, which occurs after the temperature reaches 100 °C, which is well illustrated by the decrease in the real part of complex permittivity after this temperature is reached (Figure 4a). The problem of surface-absorbed water for materials in the as-delivered state does not only apply to MgO. This issue, among others, is discussed by Polanský et al. [47] in their study on clay minerals.

From the results, it is also evident that there is no significant frequency dependence of the loss factor (Figure 4b) with the lack of visible polarization mechanisms confirming the nonpolar character of MgO. Nevertheless, there is a noticeable increase of loss factor in the higher temperature region, but this is due to the contribution of the conduction mechanism [48].

Recently, several general [49,50,51] and specific [52,53,54] review articles have focused on the synthesis and application of magnesium oxide or magnesium hydroxide nanoparticles. The aim of this review is to summarize and present the recent progress for the synthesis of magnesium oxide nanoparticles using a bottom-up approach (co-precipitation, sol–gel, solvo-/hydrothermal, combustion, and green synthesis). From this point of view, this article presents an overview of the substances and reaction conditions used together with the achieved selected parameters, such as morphology, crystalline size, and bandgap levels. This fact-finding review is complemented by the use of magnesium oxide from selected perspectives of electrical engineering applications, focusing on the modification of basic materials and primarily polymeric materials.

## 2. Synthesis of Magnesium Oxide Nanoparticles

**Figure 5:**
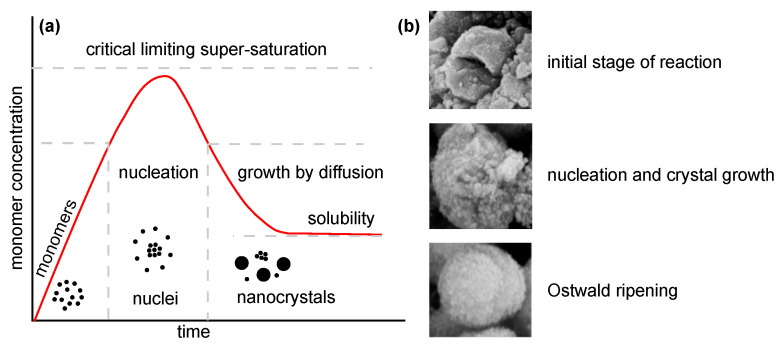
Illustration of nanoparticle growth: (**a**) LaMer model of nucleation and growth (redrawn and adapted from [64,65]) and (**b**) phases of growth (reprinted from article [66] distributed under the terms and conditions of the Creative Commons Attribution (CC BY) license).

Several approaches (Table 1) can be used for the synthesis of nanostructured MgO. In the following, insights from the most commonly used chemical and biological synthesis methods are presented (bottom-up approach) [55]. Specifically, these include sol–gel [56], solvo-/hydrothermal [57], combustion [58], co-precipitation [59] and, also, so-called green synthesis [60]. This bottom-up approach is preferable mainly because the size and morphology of the nanoparticles can be more easily controlled [61], and it is, therefore, the main topic of this review. The key aspects of this process are nucleation and crystal growth. This can be illustrated in the form of LaMer burst nucleation. Ostwald ripening [62] or coalescence [63] can be considered to describe the mechanism of further particle growth. The whole process is illustrated in Figure 5.

### 2.1. Co-Precipitation

**Figure 6:**
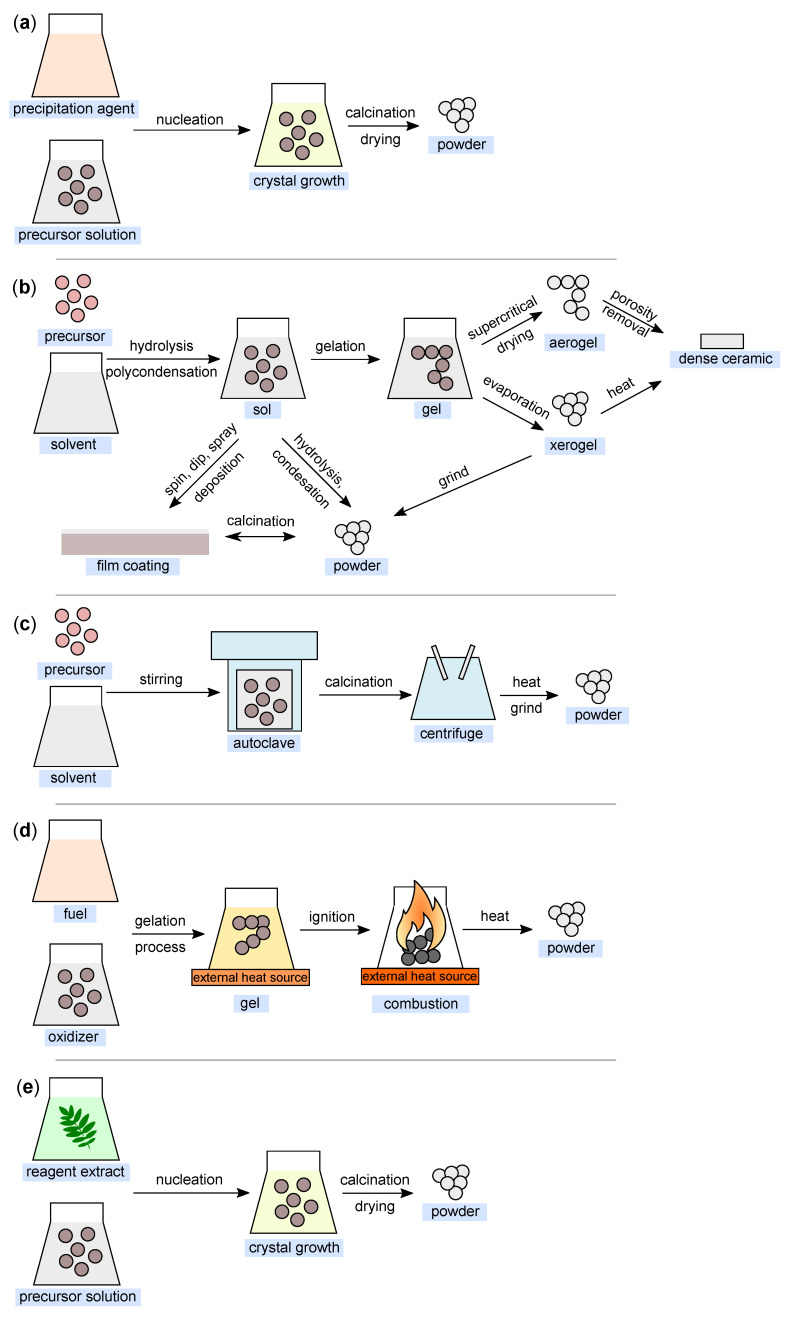
Illustration of nanoparticle synthesis using the (**a**) co-precipitation method; (**b**) sol–gel method; (**c**) hydrothermal method; (**d**) combustion method; and (**e**) green synthesis (all figures are redrawn and adapted from [118,119,120,121,122]).

This method is also widely used for the synthesis of nanoparticles (Figure 6a). It is based on the principle of precipitation and very often involves liquid-phase synthesis [67] and, less often, vapor-phase synthesis [68]. Sodium hydroxide is commonly used as the precipitating agent [69,70]. The basic principle is the homogenization of the precipitation reaction involving two processes—nucleation and nuclei growth [71].

Generally, three principles are considered: (i) single nucleation and uniform growth by diffusion; (ii) nucleation, growth, and aggregation of smaller subunits; and (iii) multiple nucleations and Ostwald ripening growth [72]. The critical solute concentration that initiates the process plays a major role in the classical process, with solute diffusion on the surface causing growth. Furthermore, it is necessary to separate these two processes. The resulting precipitate is then washed and dried.

Mashad et al. [73] prepared MgO particles using the co-precipitation method by observing the effect of different reaction conditions, such as temperature, pH, and the molar ratio of precursor (magnesium nitrate). Using the procedure presented in their work, they formed nanoparticles and nanorods with a relatively high specific surface area (nanoparticles 231 m${}^{2}$/g, nanorods 176 m${}^{2}$/g) and particle size of 50 nm. The results show that there is an effect of a template polyethylene glycol (PEG) and pH on particle morphology.

Kumar et al. [74] also used the co-precipitation method with magnesium nitrate as the precursor and ammonium hydroxide solution as the precipitating agent. This resulted in particles with an average size of about 11 nm.

Karthikeyan et al. [75] also studied the effect of PEG concentration on the properties of MgO prepared by the co-precipitation method. Magnesium nitrate was again used as precursor and sodium hydroxide as a precipitation agent. The crystallite size determined by XRD shows that using PEG is almost double compared to pure MgO (8.62 nm vs. 14.76–15.78 nm). There are also visible differences that are due to the presence of PEG, in the terms of morphology. Pure MgO had a spherical shape and PEG-modified MgO had a flake-like structure.

Frantina et al. [76] prepared MgO particles by calcination of magnesium carbonate, which they first synthesized by mixing ammonium carbonate and magnesium chloride. The XRD results show an average crystallite size of 24 nm cubic structure. The spherical morphology of the particles was determined by scanning electron microscope (SEM) with insignificant differences in particle size (an average of 50.9 nm).

Kushwaha et al. [77] synthesized MgO particles by four different methods, including the co-precipitation method (magnesium nitrate and sodium hydroxide). The results show that this chemical procedure could be used to prepare MgO particles with a 4.9 eV bandgap. The authors also reported hydrodynamic particle size within the range of 100 nm, and the crystallite size was determined to be 14.82 nm according to XRD.

Tandon and Chauhan [78] also prepared MgO nanotubes using magnesium acetate and sodium hydroxide. They determined the average crystalline size to be 34.04 nm using XRD. From the FESEM results, tubular morphology is evident, with an outer diameter of approximately 78 nm and an inner diameter of 31 nm. The authors also reported a wider bandgap compared to the previous case (5.73 eV).

### 2.2. Sol–Gel Method

The sol–gel method is one of the most fundamental approaches aimed at the formation of new material structures (primarily metal oxides and similar inorganic materials) in the presence of an inorganic precursor and an organic solvent [79]. The sol–gel approach was first introduced in the mid-19th century for the production of silica gel [80]. Metal alkoxides together with suitable solvents and reactants can form homogeneous solutions, which can then form colloidal suspensions (sol) and eventually polycondense into integrated networks (gel) [81], which are then transformed into xerogels or aerogels depending on the drying method. The illustration of the sol–gel process is shown in Figure 6b.

Mustuli et al. [82], who focused on the production of nanostructured nanoparticles witht the sol–gel method, also found that using magnesium acetate tetrahydrate together with a complexing agent in the form of oxalic acid and tertiary acid could inhibit crystal growth to achieve a thermally stable nanostructure with uniform nanoparticle size distribution.

From the same precursor and complex agent, nanoparticles were also synthesized by Sutapa et al. [83]. These researchers additionally described stress, strain, and crystal energy and achieved the formation of cubic-shape crystals, which they verified by SEM and documented the highest texture coefficient value (0.98 in the crystal plane (222)).

Others who dealt with the synthesis of MgO using the sol–gel method included Wahab et al. [84], who prepared MgO particles from magnesium nitrate together with sodium hydroxide. The procedures reported in this study led to the formation of the cubic form of MgO nanoparticles with a size of 50–60 nm.

In contrast, Boddu et al. [85] described the synthesis of MgO nanoparticles with a coralline structure when using magnesium ribbons as a precursor. In this case, a solution of magnesium methoxide was first formed, followed by hydrolysis, supercritical drying, and thermal activation. This process produced particles of the mentioned structure with dimensions of 200–300 nm.

Dercz et al. [86] analyzed the structure of a nanopowder formed from MgO xerogel using magnesium methoxide as a precursor, followed by methanol and toluene. Applying the procedure reported in this study, an average crystallite size of 7.5 nm, and a specific surface area of 138 m${}^{2}$/g was achieved.

Magnesium nitrate dispersed in distilled water was used to produce nanoparticles by Rani et al. [87]. The final particles, obtained by gel grinding and subsequent annealing, reached an average size of 60 nm, as was determined by SEM measurements.

Nanostructured MgO was also prepared by Nassar et al. [88], and in this case, a combined sol–gel combustion method was used. The authors used the aforementioned magnesium nitrate and various fuels (urea, oxalic acid, and citric acid) and found that the type of fuel had a nonnegligible effect on the crystallite size and morphology (the smallest crystallite size of *ca*. 12 nm was achieved using citric acid).

### 2.3. Solvo- and Hydrothermal Method

The solvothermal method is another of the most widely used methods for controlled crystal growth of various materials [89]. When a precursor and suitable solvent are placed in an autoclave under simultaneous exposure to higher temperature and pressure, the desired products are formed [90]. It is these reaction conditions (temperature and pressure) that allow the formation of high crystallinity materials compared to the co-precipitation method [70]. In the case of the definition of the terminology of the “solvothermal” method, a medium other than water (e.g., alcohols, or organic and inorganic solvents) is generally used as the solvent. In cases where water is used as the solvent, this method can be defined as “hydrothermal” (Figure 6c).

Devaraja et al. [91] described the properties of a nanocrystalline MgO powder that had been prepared from magnesium nitrate hexahydrate and sodium hydroxide. Their presented procedure resulted in porous magnesium oxide particles with an average crystallite size of 25 nm. Among the other reported properties of the synthesized particles was the optical energy bandgap (5.5 eV).

On the other hand, Al-Hazmi et al. [92] synthesized nanofibers by a direct reaction of magnesium acetate and urea. These fibers had an average crystallite size of 6 nm corresponding to their diameter, while their length was determined by a transmission electron microscopy (TEM) measurement of approximately 10 nm.

In the work [93], Ding et al. describe the synthesis of rod-like and tube-like magnesium hydroxide. Subsequently, MgO particles were synthesized by thermal decomposition. The author’s findings show that crystallite size, shape, and structure can be relatively easily controlled using the hydrothermal method. The authors used magnesium powder, magnesium sulfate, or magnesium nitrate hexahydrate as the base material for the synthesis. They realized several morphologies (rod-like, lamellar, and needle-like) due to different experimental conditions. The resulting particles had dimensions ranging from 20 to 600 nm with a specific surface area higher than 100 m${}^{2}$/g.

The base material in the form of magnesium powder was also used by Rukh et al. [94]. Hydrogen peroxide and de-ionized water were used as the reaction medium in this study. In this route, the authors realized MgO nanoparticles with a crystallite size of 18 nm.

Other realized forms of nanostructured MgO include nanoplates, the synthesis of which is described by Duong et al. [95]. The authors also used magnesium nitrate hexahydrate, distilled water, and various morphology controlling agents such as polyethylene glycol (PEG), cetyltrimethylammonium bromide (CTAB), or sodium dodecyl sulfate (SDS). Most interestingly, the MgO prepared by the hydrothermal method in combination with the surfactant SDS had the highest specific surface area (126 m${}^{2}$/g) and achieved the desired disc morphology (diameter 40–60 nm, thickness 5 nm).

### 2.4. Combustion Method

The combustion method (Figure 6d) is a method frequently used for the synthesis of metal oxide nanoparticles, mainly due to its efficiency and low-cost [96]. It can be based on two different approaches. The first is the so-called self-propagating synthesis and the second is the volume combustion synthesis [97]. In the case of self-propagating synthesis, it involves spontaneous redox reactions ignited by an external source that takes place between the precursor (oxidizer) and the reductant (fuel) mixed at the molecular level in solution, with the formation of solid products occurring without any further input energy [98]. In the second case, the entire sample is heated until the reaction is initiated in its entire volume. This method of preparation is more difficult to control and is recommended especially for weak exothermic reactions that require preheating before ignition [99].

Balakrishnan et al. [38] synthesized MgO using the solution combustion method. They used magnesium nitrate as an oxidizer and urea as fuel. Using this method, they produced MgO with a cubic structure and crystallite size around 22 nm as seen from the XRD results. Through SEM analysis, the authors found that the particles are spherical and their size is uniform. Interestingly, compared to other studies, the synthesized particles have a bandgap of only 2.9 eV.

The same starting materials were used for the synthesis of MgO by Rao et al. [100]. The aim of their efforts was, among other things, to verify the effect of the fuel to oxidizer ratio. From the results, it is evident that with a higher fuel ratio, the synthesized particles have a higher crystallite size (18–53 nm) except in the case of 0.75 ratios. This can be attributed to changes in the ignition temperature, burn rate, or enthalpy.

Ranjan et al. [101] used a modification of this process in which glycine was used as fuel, and the precursor was magnesium nitrate. From the XRD results, they determined the crystallite size to be 20.76 nm.

Therami et al. [102], on the other hand, used citric acid as fuel. The authors investigated the effect of the proportion of this acid on the selected parameters. The most significant changes obtained were the increased bandgap, from 4.72 to 5.35 eV (higher ratio and higher bandgap), decreased particle size, from 35 to 20 nm (higher ratio and smaller size), and changes in morphology (flake-like, vacuolar, and flower-like).

Kumar et al. [103] synthesized MgO from magnesium nitrate solution and parthenium plant extract. Analysis of the effect of the amount of fuel on the bandgap width (5.3–5.45 eV) and crystallite size (27–35 nm) was the main goal of their study; however, the differences were not as significant as they were in the previous case.

### 2.5. Green Synthesis

The so-called green synthesis is a modern approach to nanoparticle production, whereby there are no (or minimal) requirements for reaction conditions (high pressure, temperature, and energy) and no toxic chemicals are used [104,105]. The aim is to minimize the produced waste and to establish sustainable development in this field [106], as nontoxic reagents can be used in this production method. These can be various agents ranging from plant extracts [107] and bacterial strains [108] to enzymes and vitamins [53]. Double distilled water is most commonly used as the extraction medium in this process. In simplified terms, the process can be considered to comprise three fundamental stages: (i) activation, (ii) growth, and (iii) process termination. A simplified process diagram is shown in Figure 6e.

Suresh et al. [109] used an extract of *Nephelium lappaceum* L. and double distilled water for green synthesis, whereby magnesium nitrate was used as a precursor. They verified the cubic structure of MgO by the investigations that were carried out, and the average crystallite size was determined as 55 nm, which agreed very well with the SEM measurements (grain size 60–70 nm).

Vargheese and Vishal [110] synthesized MgO by using *Trigonella foenum-graecum* extracted in double distilled water using magnesium nitrate as a precursor. The average crystalline size determined from XRD was around 14 nm. It is evident from the SEM measurements that the particles prepared in this way had a mixture of fine, spherical structures.

Younis et al. [111] used *Rosa floribunda* powder, which they dispersed in double-distilled water, and—as in earlier cases—magnesium nitrate was used as a precursor. The results show a cubic structure of high purity, and the nanoparticle size, as determined using high-resolution TEM, was around 10 nm.

The synthesis of *Rosmarinus officinalis* L. with bulk MgO was studied by Abdallah et al. [112]. The resulting nanoparticles contained minimal impurities and had a hexagonal crystal structure, unlike in the previous cases. The average particle size described in this study was 8.8 nm.

Khan et al. [113] used *Dalbergia sissoo* extract and magnesium nitrate as a precursor. In their study, the authors focused on describing several aspects (extract concentration, precursor, and pH) affecting the bandgap size and photocatalytic activity. The cubic structure of MgO is evident from the results. In addition to this, SEM measurements were also performed, demonstrating a particle size of around 50 nm.

The use of *Saussurea costus* biomass was the focus of the work by Amina et al. [114], in which magnesium nitrate was used as a precursor. The results show that the authors were able to produce particles with a cubic structure and a size of about 30 nm using this procedure.

On the other hand, MgO particles with dimensions less than 20 nm were formed by phyto-assisted synthesis by Sharma et al. [115]. They used *Swertia chirayaita* as the reactant and magnesium nitrate as the precursor. The SEM results reported in their study show the spherical shape of the particles with slight variation in shape.

Using magnesium chloride as a precursor and *Moringa oleifera* as a reagent, Fatiquin et al. [116] attempted to synthesize MgO. Their efforts resulted in particles with a crystalline size of around 21 nm. These particles, according to TEM, exhibited a cubic structure and range in size between 2 and 50 nm.

Nguyen et al. [117] synthesized MgO particles from extracts of *Tecoma stans* L. (flower, bark, and leaf) and a magnesium nitrate precursor. The resulting particles had a spherical or hexagonal morphology depending on the used extract, with the average crystal size ranging from 20 to 50 nm. The authors determined that the flower extract was the most promising, mainly due to the high absorption capacity of the synthesized particles.

**Table 1:** Individual substances and reaction conditions for nanostructural MgO synthesis via different methods based on the bottom up-approach.

Co-Precipitation Method							
Precursor	Precipitation agent	Reaction temperature	Calcination temperature	Calcination time	Size	Expected application	Ref.
Mg(NO${}_{3}$)${}_{2}$	NH${}_{3}$H${}_{2}$O	60; 70; 80 °C	550 °C	2 h	50 nm	-	[73]
Mg(NO${}_{3}$)${}_{2}$	NH${}_{4}$OH	100 °C	600 °C	4–6 h	11 nm	antibacterial	[74]
Mg(NO${}_{3}$)${}_{2}$	NaOH	room	500 °C	4 h	14–16 nm	antibacterial	[75]
(NH${}_{4}$)${}_{2}$CO${}_{3}$	MgCl${}_{2}$	80 °C	550 °C	4 h	24 nm	-	[76]
Mg(NO${}_{3}$)${}_{2}$	NaOH	-	440 °C	4.5 h	*ca.* 15 nm	catalyst	[77]
Mg(NO${}_{3}$)${}_{2}$	NaOH	room	room	-	78 nm	antibacterial	[78]
**Sol–Gel Method**							
Precursor	Solvent	Gel drying temperature	Calcination temperature	Calcination time	Size	Expected application	Ref.
Mg(CH${}_{3}$COO)${}_{2}$	C${}_{4}$H${}_{6}$O${}_{6}$	-	600 °C	6 h	-	-	[82]
Mg(CH${}_{3}$COO)${}_{2}$	C${}_{2}$H${}_{2}$O${}_{4}$	200 °C	950 °C	6 h	-	-	[83]
Mg(NO${}_{3}$)${}_{2}$	NaOH	300 °C	500 °C	2 h	50–60 nm	adsorber	[84]
Mg(OCH${}_{3}$)${}_{2}$	CH${}_{3}$OH; C${}_{7}$H${}_{8}$	-	500 °C	5 h	200–300 nm	-	[85]
Mg(OCH${}_{3}$)${}_{2}$	CH${}_{3}$OH; C${}_{7}$H${}_{8}$	60 °C	450 °C	-	*ca.* 8 nm	-	[86]
Mg(NO${}_{3}$)${}_{2}$	dH${}_{2}$O	150 °C	500 °C	2 h	60 nm	-	[87]
Mg(NO${}_{3}$)${}_{2}$	C${}_{6}$H${}_{8}$O${}_{7}$; C${}_{2}$H${}_{2}$O${}_{4}$;	350 °C	550; 800 °C	2 h	12 nm	catalyst	[88]
**Solvo- and Hydrothermal Method**							
Precursor	Solvent	Autoclave temperature	Calcination temperature	Calcination time	Size	Expected application	Ref.
Mg(NO${}_{3}$)${}_{2}$	NaOH	130 °C	400–800 °C	2 h	25 nm	-	[91]
Mg(CH${}_{3}$COO)${}_{2}$	NH${}_{2}$CONH${}_{2}$	180 °C	600 °C	1 h	6 nm	antibacterial	[92]
Mg(NO${}_{3}$)${}_{2}$	NaOH	80 °C	280–450 °C	1; 2; 2 h	50 nm	catalyst	[93]
MgSO${}_{4}$	NH${}_{3}$H${}_{2}$O; en-H${}_{2}$O	180 °C	280–450 °C	1; 2; 2 h	100–200 nm	catalyst	[93]
Mg	H${}_{2}$O${}_{2}$	220 °C	-	-	18 nm	antibacterial	[94]
Mg(NO${}_{3}$)${}_{2}$	NaOH	100 °C	500 °C	4 h	40–60 nm	adsorber	[95]
Oxidizer	Fuel	Ignition temperature	Calcination temperature	Calcination time	Size	Expected application	Ref.
Mg(NO${}_{3}$)${}_{2}$	NH${}_{2}$CONH${}_{2}$	70-80 °C	500 °C	3 h	22 nm	adsorber	[38]
Mg(NO${}_{3}$)${}_{2}$	NH${}_{2}$CONH${}_{2}$	100 °C	300 °C	2 h	18–53 nm	-	[100]
Mg(NO${}_{3}$)${}_{2}$	NH${}_{2}$CH${}_{2}$COOH	170 °C	600 °C	2 h	*ca.* 21 nm	fuel additive	[101]
Mg(NO${}_{3}$)${}_{2}$	C${}_{6}$H${}_{8}$O${}_{7}$	100 °C	400 °C	15 min	20–35 nm	antibacterial	[102]
Mg(NO${}_{3}$)${}_{2}$	Parthenium	400 °C	-	-	27–35 nm	photocatalyst	[103]
	extract						
**Green Synthesis**							
Precursor solution	Reagent extract	Reaction temperature	Calcination temperature	Calcination time	Size	Expected application	Ref.
Mg(NO${}_{3}$)${}_{2}$	*Nephelium*	80 °C	450 °C	-	55 nm	-	[109]
	*lappaceum*						
Mg(NO${}_{3}$)${}_{2}$	*Trigonella*	80 °C	600 °C	4 h	14 nm	antibacterial	[110]
	*foenum-graecum*						
Mg(NO${}_{3}$)${}_{2}$	*Rosa floribunda*	90 °C	-	-	10 nm	antibacterial	[111]
Bulk MgO	*Rosmarinus*	70 °C	-	-	*ca.* 9 nm	antibacterial	[112]
	*officinalis*						
Mg(NO${}_{3}$)${}_{2}$	*Dalbergia sissoo*	30–70 °C	-	-	50 nm	photocatalyst	[113]
Mg(NO${}_{3}$)${}_{2}$	*Saussurea costus*	80 °C	450 °C	3 h	30 nm	photocatalyst	[114]
Mg(NO${}_{3}$)${}_{2}$	*Swertia chirayaita*	55 °C	400 °C	4 h	<20 nm	antibacterial	[115]
MgCl${}_{2}$	*Moringa oleifera*	90 °C	600 °C	5 h	*ca.* 21 nm	antibacterial	[116]
Mg(NO${}_{3}$)${}_{2}$	*Tecoma stans*	90 °C	550 °C	6 h	20–50 nm	adsorber	[117]

## 3. Selected Technical Applications of Magnesium Oxide

Since the application uses of nanostructured magnesium oxide are very broad, ranging from agriculture to civil engineering, the main focus of this paper is on applications related to utilizing the properties of magnesium oxide in electrotechnical applications. In this category, the focus has been mainly on the areas of sensor technology, modification of the insulation properties of polymer insulation systems, and optimization of polymer-based electrolytes for batteries.

### 3.1. Sensors Based on MgO

Sensors and various types of monitoring are an integral part of everyday life. Due to this fact, efforts are being made to improve their sensitivity and stability as well as minimize their dimensions as much as possible [123]. In the following, examples of the application of nanostructured magnesium oxide in interaction with various elements and substances for their detection are presented.

Among the most basic environmental sensors are humidity sensors. Murkute et al. [124] presented a Zn_1-x_Mg_x_O-based humidity sensor (hydrothermal method) with excellent optical properties. The synthesized material had a nanorod morphology with crystallite sizing ranging from about 20 to 30 nm depending on the percentage of magnesium oxide composition. In principle, this chemoresistive sensor is based on the autoionization of water vapor on the surface of synthesized nanorods (passivation of Zn^+^ and O_2_^−^ defect states using H^+^ and OH^−^ dissociated ions) and the corresponding change in impedance. The proposed sensor exhibited a very high sensitivity of 1.503% RH for the change in RH and a response time of about 8 min.

Shukla et al. [125] were the next to implement a MgO-based humidity sensor.Their work resulted in a thin film (coating) formed by MgO particles on the surface of a glass optical fiber with a U-shape. The synthesized particles (sol–gel) had a grain size of 50–60 nm. Simplistically, this sensor also works on the principle of water vapor absorption in the MgO thin film, resulting in changes in the refractive index and output power.

Sensor elements are also used to detect hazardous gases in the environment. Across the sensor spectrum, one of the most prominently studied gases is hydrogen sulfide [126], which can occur as a minor component of biogas. El Shamy [127] presented the design of his C_Dots_@MgO sensor working on the Schottky junction principle. The hydrothermal approach was used to synthesize MgO, and microwave heating was used to synthesize C_Dots_. The realized particles did not exceed 30 nm in size and had spherical morphology. The subsequent spin coating on the p-Si wafer was used to deposit the formed C_Dots_@MgO nanoparticles. The work showed a significant change in the Schottky barrier height, resistivity, and conduction current due to the interaction of H_2_S and oxygen adsorbed on MgO surface (electron donation of MgO), and this phenomenon was greatly enhanced by the decoration of C_Dots_ on the MgO surface. From the performed measurements, the high stability, and repeatability at different H_2_S concentrations (10–120 ppm) are clear.

In the context of biogas, it is also necessary to monitor the methane concentration in terms of biogas deflagration [128]. Sertel et al. [129] developed a methane sensor based on the synthesis of MgO:TiO_2_ thin films, which were deposited on platinum electrodes using a classical photolithography technique. The grain size of the nanoparticles was in the range of 23–28 nm. The sensor thus formed showed a sensitivity of 33.1–43.5%, and response times of 6, 9, and 8 s were observed depending on the depositing conditions (substrate temperature and annealing temperature). The results show that the MgO doped TiO${}_{2}$ film had decreased resistance when interacting with the testing gas. The authors of this study attributed this fact to the effect of the p-type conductivity of MgO.

An interesting application of a thin film MgO-based sensor was described by Tao et al. [130]. The proposed sensor was capable of high-sensitivity detection of 2-methoxyethanol and 2-ethoxyethanol (known as ethylene glycol ethers) vapors, which are abundantly found in various solvents, colorants, and stabilizers in the dye industry. A sol–gel method was used to form the MgO particles, followed by immersion of the ceramic substrate in the precursor solution. The final film thickness was estimated as 230 nm. In this case, it is assumed that photoemission activity occurs due to consecutive chemical reactions. Specifically, this involves catalytic oxidation of the gases of interest with oxygen together with the electronic excitation of methoxy acetaldehyde or ethoxy acetaldehyde and their absorption and subsequent return to a ground state. The detection limit of the proposed sensor (1 and 1.4 ppm) is below the permissible concentration (8.8 and 8.9 ppm) of selected ethylene glycol ethers, and thus the sensor is applicable for real-time monitoring of workplace conditions with a response time of about 5 s.

The requirement for monitoring hazardous substances in the environment may also be required for liquids, e.g., water. Kokulnathan et al. [131] exploited the electrochemical activity of MgO decorated on graphene oxide (GO) toward the detection of nitrobenzene in water. The hydrothermal approach was used for the synthesis of MgO particles (spherical morphology). Hummers’ method was used for the synthesis of GO (multilayered structure). The decoration of MgO on GO was carried out by one-pot synthesis. Due to the synergistic effect of GO and MgO, fast electron transfer leading to superior electrochemical properties can be achieved. The detection limit of 0.01 µM of nitrobenzene can be highlighted from the results.

### 3.2. Electrical Insulating Materials Filled with MgO

Magnesium oxide implementation areas of interest include dielectric applications. In these cases, MgO nanoparticles are incorporated in order to modify not only dielectric parameters, such as internal resistivity and dielectric loss as well as mechanical or thermal properties. A considerable amount of work has been conducted in this area. If the focus is on improving the electrical insulation parameters of commonly used types of insulation, two basic directions can be defined. Modifications of thermoplastic materials (mainly cable insulation) and thermosetting materials (mainly epoxy-based resins).

Pallon et al. [132] focused on the modification of polyethylene for high voltage DC cables. The authors were able to achieve a conductivity reduction within two orders of magnitude by incorporating MgO with hexagonal shape (co-precipitation method) with an average nanoparticle size of 70 nm. This is particularly the case at lower filling levels (less than 3 wt.%) since, in these cases, at least partial dispersion of the nanofiller is maintained. Moreover, changes in the presence and character of electron traps play a role in this case. At higher concentrations, there is a significant effect of agglomeration and surface-absorbed water, and these aspects contribute to the deterioration of the insulating properties of the composite. By using hydrocarbon functional silsesquioxane coatings, a relatively good level of particle dispersion was subsequently achieved. For uncoated particles, some agglomerates reached up to the order of microns.

The issue of cable insulation, or the possibility of improving it, was addressed by Paramane et al. [133]. In their case, the effect of MgO on selected dielectric parameters, such as electrical strength, conductivity, or space charge, was studied during a thermal aging experiment. The results show that there was a 20% improvement in electrical strength, an order of magnitude decrease in electrical conductivity, and a modification of the space charge distribution due to structural changes, such as an increase in deep traps or changes in material crystallinity. The SEM results show very good dispersion of the particles.

Lin et al. [134] used polypropylene (PP) as the matrix because of its easier recycling due to its thermoplasticity. In their work, the authors focused on studying the effect of MgO in combination with aluminum nitride (AIN) coated by γ-methacryloxypropyltrimethoxysilane. Their investigation confirms the above findings of an increase in electrical strength and a decrease in material conductivity due to the incorporation of surface-treated MgO. Moreover, the results show that MgO combined with AIN did not lead to improved insulating properties compared to pure PP.

An even more detailed analysis of the incorporation of MgO in PP was performed by Hu et al. [135], who also focused on the effect of its surface treatment. The targets of their study were silane coupling agents with different alkyl chain groups (methyl—C1, propyl—C3, octyl—C8, and octadecyl—C18). Interestingly, all the prepared composites had better electrical properties than pure PP. This can be attributed to the increase in the number of deep traps due to the presence of alkyls. Based on all the results, the PP-MgO-C8 combination was reported to be the most favorable, using 3 wt.% of MgO.

Not only the electrical strength and conductivity of the material but also the discharge activity can be affected by the addition of MgO (0.5 wt.%). Zhu et al. [136] confirmed that the level of discharge activity in MgO/XLPE was lower than that in pure XLPE. On the other hand, the polymer that was modified in this way was shown to have a lower resistance to electric tree growth in the presence of a strongly inhomogeneous electric field. This is mainly due to the change in the ratio of the amorphous (increase) and crystalline (decrease) phases of the polymer due to the incorporation of magnesium oxide. This subsequently leads to an increase in the electron bombardment of the molecular chains and a gradual erosion of the internal structure.

As already mentioned, the presence of nanoparticles also affects the mechanical properties. Kiaei et al. [137] also verified this for MgO. They found that, at a concentration of 3%, there was an increase in flexural strength and modulus. For a higher level of filling, there is a deterioration in these properties. In addition to the mechanical properties, the authors reported that the higher the concentration of MgO, the lower the burning rate and release heat, which is due to the MgO layer postponing oxygen penetration into the polymer base.

In the case of magnesium oxide incorporation into epoxy resin, several research studies have been conducted by Hornak et al. [46,138,139,140,141]. From these studies, it is evident that MgO shows good miscibility with the most common type of epoxy-based on bisphenol A epichlorohydrin in its different variations (single and double component, heat and cold cure) and at lower concentrations (close to 1 wt.%), which results in a reduction in internal conductivity within an order of magnitude and can especially be attributed to the reduction of the trapped charge inside the material due to the changes in electron trap density and depth.

However, there is a slight increase in the loss number with increasing MgO concentration [140], especially in the lower frequency region, where the effect of impurities on the conductivity character becomes apparent. Studies [46,141] investigated the effect of a silane-based coupling agent (γ-Glycidyloxypropyl)trimethoxysilane). The results show that using the modified nanoparticles, there is a further reduction in the conductivity of the material due to the better dispersibility in polymer matrix.

In the subsequent final study [139], the polyethylene napthalate film covered by epoxy resin with dispersed MgO was investigated, and the resulting material was compared with commonly used dielectric materials for insulating of rotating machines. The results show that the designed material achieves qualitatively improved dielectric properties especially with respect to the dielectric strength, volume resistivity, and dielectric losses.

A comprehensive study on the behavior of MgO epoxy composite in terms of electrical, thermal, and mechanical properties was published by Peddamallu et al. [142]. Their investigation shows that the higher the MgO concentration the higher the relative permittivity of the composite. There was also a significant improvement in tensile and flexural strength, and the results of the dynamic mechanical analysis show an increase in storage modulus with the increasing concentration of MgO nanoparticles. The glass transition temperature also increases with increasing MgO concentration. This could be due to the decrease in chain mobility of polymer chains resulting from the presence of filler.

Another relatively comprehensive study was presented by Ge et al. [143]. In their work, the authors characterized, among other things, the changes in thermal conductivity of the composite material as a function of the filling level. This shows that the higher the filling concentration is, the higher the thermal conductivity of a given composite. This can be attributed to the fact that the MgO particles and the polymer matrix interacted with each other at higher filling content to form parallel structures, which increased the thermal conductivity of the whole. This increase may be of interest for applications where higher heat dissipation is required. However, the study further shows and confirms the results presented in [46], that, at filling levels higher than 1 wt.%, the insulation parameters (dielectric strength and volume resistivity) deteriorate.

Thermal conductivity was also addressed by Wereszczak et al. [144]. They, on the other hand, took the route of higher concentrations (49–56 wt.%). Their results show that when the base epoxy matrix was doped with the maximum filling level, an up to tenfold increase in thermal conductivity (>3 W/mK) was achieved.

### 3.3. Polymer-Based Electrolytes Doped with MgO

Polymer electrolytes are used due to their flexibility, low weight, and easy processability [145]. They can be most commonly divided into solid polymer electrolytes, gel polymer electrolytes, and composites [146]. The origin of the electrolyte can affect parameters, such as the ionic conductivity, electrochemical stability windows, or mechanical resistance. Hence, there is space for material improvement.

Masoud et al. [147] focused on the synthesis of MgO nanoparticles with their subsequent incorporation into the electrolyte base. They used the solution combustion method for synthesis, and the resulting nanostructure had a crystallite size of 40 nm. The authors reported a concentration of 6 wt.% MgO and 25 wt.% lithium trifluoromethanesulfonate (LTF) in the base polymer blend (polyvinylidene fluoride-co-hexafluoropropene) as the most optimal. In this combination, the electrolyte showed electrochemical (decomposition voltage = 3 V) and mechanical stability (elongation at break of 5.7%), and AC-ionic conductivity (8.78 × 10^−5^ S/cm). The increase in AC-ionic conductivity could be attributed to the decreased polymer crystallinity and the creation of a conducting pathway by the nanofiler.

A polymer electrolyte with incorporated MgO was also studied by Wu et al. [148]. In their case, the electrolyte base consisted of polyurethane/polyvinylidene fluoride). The most optimal concentration in terms of electrochemical (window 4.7 V) and mechanical stability (elongation 91.7%) and ionic conductivity (4.6 × 10^−3^ S/cm) was 7 wt.% MgO. When higher quantities were incorporated, the motion of mobile ions was reduced.

MgO particles prepared by green synthesis were used by Zaky et al. [149]. In this case, the authors focused on determining the optimum concentration of MgO in polyethylene glycol with Mg salts. The effect of gamma irradiation was also investigated. In terms of electrochemical stability (electrochemical window 4.4 V), the inclusion of 30 mL of nanoparticles without irradiation was the most optimal. In the case of irradiated electrolytes, the highest electrochemical stability (window 3.9 V) was found using 20 mL. In terms of electrical conductivity, the increase is mainly due to the space charge layer at the interface between the filler and the polymer assisting Mg^2+^ ion transport in the amorphous polymer electrolyte. From observing the effect of irradiation, it can be concluded that the conductivity increases with increasing radiation doses (except for when including 30 mL).

## 4. Conclusions

Magnesium oxide, and specifically its nanostructured form, is a widely demanded and used material from the group of simple metallic oxides. Many methods can be used to synthesize MgO. Current trends show a significantly increased interest in green synthesis, particularly concerning the elimination of chemicals and produced waste. With the focus is on shape, there is the possibility to synthesize MgO of very different shapes. Most often, these are particle-like structures, but rod-like, flat-like, or tubular structures can also be found. In these cases, the size of the MgO was in the order of tens of nanometers, and the specific surface area was larger than 100 m${}^{2}$/g. Magnesium nitrate was the most commonly used precursor.

As far as applications are concerned, there is a considerable multidisciplinary range. However, this publication aimed to summarize the use of nanostructured MgO in selected engineering and technological applications. The properties of MgO can be exploited, for example, in sensor technology (environmental, gas, or liquid sensors) or the modification of cable insulation for DC distribution grids. These findings are of great interest for the present developmental efforts in the field of DC distribution that are currently underway.
